# Autoimmune Neuromuscular Disorders

**DOI:** 10.2174/157015911796558000

**Published:** 2011-09

**Authors:** Jessica Kraker, Saša A Živković

**Affiliations:** 1 Department of Neurology, University of Pittsburgh Medical Center, Pittsburgh, PA, USA; 2 VA Pittsburgh Healthcare System, Pittsburgh, PA, USA

**Keywords:** Autoimmune, neuromuscular, neuropathy, myositis, myasthenia gravis, treatment.

## Abstract

Autoimmune neuromuscular disorders affecting peripheral nerves, neuromuscular junction or muscle have a wide clinical spectrum with diverse pathogenetic mechanisms. Peripheral nervous system may be targeted in the context of complex immune reactions involving different cytokines, antigen-presenting cells, B cells and different types of T cells. Various immunomodulating and cytotoxic treatments block proliferation or activation of immune cells by different mechanisms attempting to control the response of the immune system and limit target organ injury. Most treatment protocols for autoimmune neuromuscular disorders are based on the use of corticosteroids, intravenous immunoglobulins and plasmapheresis, with cytotoxic agents mostly used as steroid-sparing medications. More recently, development of specific monoclonal antibodies targeting individual cell types allowed a different approach targeting specific immune pathways, but these new treatments are also associated with various adverse effects and their long-term efficacy is still unknown.

## INTRODUCTION

Autoimmune neuromuscular disorders affecting peripheral nerves, neuromuscular junction or muscle have a wide clinical spectrum with diverse pathogenetic mechanisms [1-3]. Peripheral nervous system may be targeted in the context of post-infectious immune reaction, paraneoplastic syndromes and often we do not find any triggering or preceding events (Table **1**). Pathogenetic mechanisms involve complex interactions between antigen-presenting cells, B cells and different types of T cells. Various immunomodulating and cytotoxic treatments block proliferation or activation of immune cells by different mechanisms attempting to control the response of the immune system. Most treatment protocols for autoimmune neuromuscular disorders are based on the use of IVIG, corticosteroids and plasmapheresis, with cytotoxic agents mostly used as steroid-sparing medications. More recently, development of specific monoclonal antibodies targeting individual cell types allowed a different approach targeting specific immune pathways, but only time will tell whether these new treatments are as effective and safe as ”classic” regimens [4].

## A. AUTOIMMUNE NEUROMUSCULAR DISORDERS

### 1. Autoimmune Neuropathies

Neuropathies with immune-mediated etiologies may manifest acutely or chronically, resulting in primary demyelination or axon loss. Similarities between peripheral nerve glycolypids and myelin proteins with various infectious agent components may result in molecular mimicry and trigger an immune response cross-reacting with peripheral nerves. In animal model of experimental autoimmune neuritis (EAN), immunization with peripheral nerve components leads to autoimmune reaction and peripheral nerve inflammation resembling Guillain Barre syndrome and allowing the study of different autoimmune and inflammatory pathways [2]. 

#### 1.1. Guillain Barre Syndrome

Guillain-Barre Syndrome (GBS) is an acquired inflammatory polyradiculoneuropathy with an annual incidence of 1-2/100,000 [5]. Up to two-thirds of cases may have an antecedent flu-like illness or gastroenteritis triggering the immune response. Complex pathophysiology of nerve injury in GBS mostly involves humoral immunity mechanisms targeting peripheral nerve antigens. Elevated titers of anti-nerve antibodies are frequently found but their presence usually has limited clinical significance. In western countries, most patients are affected by demyelinating variant of GBS (acute inflammatory demyelinating polyneuropathy, AIDP), while in eastern Asia axonal type predominates (acute motor axonal neuropathy; AMAN) [5]. Differences between AIDP and AMAN were attributed to the variations of the primary targets of the immune response. In AIDP, histopathologic features of demyelination are also accompanied by secondary axon loss.

Clinically, GBS manifests as an acute peripheral neuropathy with symmetric weakness reaching a peak by 4 weeks from onset, hyporeflexia or areflexia, and cytoalbuminemic dissociation in the cerebrospinal fluid (CSF) with an elevated protein content and normal cell count [5]. Ventilatory failure and dysautonomia are relatively common. Electrodiagnostic studies demonstrate evidence of demyelination (AIDP) or axon loss (AMAN).

Standard treatments of GBS include IVIG or plasmapheresis which both reduce the need for mechanical ventilation, and increase the speed of recovery [6]. Guidelines of American Academy of Neurology recommend IVIG and plasmapheresis as equivalent treatments used within 4 weeks from onset of symptoms [7]. Combination of IVIG and plasmapheresis or monotherapy with corticosteroids are not recommended [7].

Less frequent variants of GBS include (a) Miller-Fisher syndrome manifesting with ophthalmoplegia, ataxia and areflexia and elevated titers of GQ1b antibodies; (b) sensory GBS and (c) acute dysautonomic neuropathy (“small fiber GBS”). Miller-Fisher syndrome is mostly self-limited condition with good prognosis and there are no controlled studies of treatment [8]. Few anecdotal reports suggest benefits of immunotherapy with IVIG.

#### 1.2. Chronic Inflammatory Demyelinating Polyradiculoneuropathy (CIDP)

Chronic inflammatory demyelinating polyradiculoneuropathy (CIDP) is also demyelinating inflammatory polyneuropathy with slower progression than AIDP and is defined by nadir of weakness occurring at 8 weeks or longer after the onset of symptoms [9]. The clinical features of CIDP include progressive or stepwise chronic symmetric, proximal greater than distal weakness, less prominent sensory loss and paresthesias and hyporeflexia. Cranial nerve involvement, respiratory involvement, and dysautonomia occur far less frequently than with AIDP. Variants of CIDP include Lewis-Sumner syndrome (multifocal acquired demyelinating sensory and motor neuropathy; MADSAM) and distal acquired demyelinating sensory and motor neuropathy (DADS) [10-12]. Pathophysiology of nerve injury in CIDP is still not well understood. However, a combination of humoral and cell-mediated immune mechanisms leading to demyelination and secondary axon loss invokes the similarity with multiple sclerosis in CNS. Elevated titers of autoantibodies targeting peripheral nerve glycolipids and myelin are also found in CIDP, but the clinical significance is limited. As opposed to AIDP, preceding infections are also much less common.

Most patients with CIDP respond initially to treatment, but the relapse rate may be as high as 50% within first four years from the onset of therapy. Treatment with corticosteroids or IVIG should be considered as a first line therapy, and plasmapheresis may be associated with higher rate of relapses [13]. Steroid-sparing agent should be considered to avoid side-effects related to chronic corticosteroid use. Long-term follow-up studies of CIDP showed that at 5 years, 23% of patients are in continuous remission off medications, 60% are in partial remission and 13% may remain severely impaired [14]. Second line treatments include azathioprine and cyclophosphamide [15, 16].

#### 1.3. Vasculitic Neuropathy

Vasculitic neuropathy is usually a fairly “aggressive” condition which may lead to severe disability and requires prompt and accurate diagnosis. Vasculitis may present in the context of systemic vasculitis or may affect peripheral nerves only [17, 18]. Clinically, nerve vasculitis typically presents with a painful asymmetric sensorimotor polyneuropathy with predominant axon-loss features [18]. Nerve injury stems from injury of nerve vessels and resulting ischemic damage. As treatments for vasculitis carry significant morbidity, histopathological confirmation of the diagnosis is preferred prior to treatment.

In nonsystemic vasculitic neuropathy, corticosteroid monotherapy is usually considered the first choice, although combination with cyclophosphamide may be more effective [17]. Corticosteroids are also mainstay of therapy for systemic vasculitis and are usually used in combination with other immunosuppressives [18]. In secondary vasculitides, one of our main goals is to remove the trigger (or cause) of vasculitis. We also have to treat infections which may have triggered vasculitis and in viral-associated vasculitides, chronic immunosuppression is usually avoided. Plasma exchange may be considered for treatment of viral-associated vasculitides, but the benefits remain uncertain [19].

#### 1.4. Paraproteinemic Neuropathy

Monoclonal gammopathies are a heterogeneous group of disorders characterized by the clonal proliferation of plasma cells and production of monoclonal M-proteins (paraproteins). Monoclonal gammopathy occur in the context of hematologic malignancies or isolated as monoclonal gammopathy of undetermined significance (MGUS) [20]. The finding of M-proteins in the serum is common and increases with age, occurring in up to 1% of healthy adults over the age of 25 and 3% of patients over the age of 70. Monoclonal gammopathies have been described in up to 10% of patients with cryptogenic peripheral neuropathy [21]. Conversely, in patients with MGUS up to one third will have peripheral neuropathy. Most of the patients with MGUS-related neuropathy have monoclonal IgM immunoglobulins. Over time an increasing proportion of patients with MGUS may develop hematologic malignancies, especially multiple myeloma [22]. Other disorders associated with paraproteinemic neuropathy include Waldenstrom’s macroglobulinemia, osteosclerotic myeloma, primary amyloidosis (light chain), lymphoma or leukemias [20]. Almost half of the patients with an IgM M-protein and neuropathy will have elevated titers of antibodies against myelin-associated-glycoprotein (MAG). Typically, anti-MAG antibody-associated neuropathy presents as an insidious, distal, predominantly sensory neuropathy with some distal weakness. Peripheral neuropathy associated with anti-MAG antibodies is commonly treatment-resistant, but recent reports suggest that rituximab may be helpful [23, 24]. Potential risks and benefits of treatment with rituximab should be considered including slowly-progressive nature of the condition, long-term disability and potential risk of opportunistic infections [25, 26]. Patients with paraproteinemic neuropathy associated with IgG/IgA monoclonal proteins frequently benefit from plasmapheresis [27]. CIDP associated with monoclonal gammopathy (usually IgA/IgG) responds to therapy in the same fashion as "idiopathic" CIDP [28].

#### 1.5. Paraneoplastic Neuropathy

Paraneoplastic neuropathy is most commonly associated with small cell lung cancer and hematologic malignancies. These malignancies may incite an autoimmune response targeting antigens in different tissues (including peripheral nerve) without direct invasion of peripheral nervous system by malignancies. Titers of paraneoplastic antibodies are frequently elevated including anti-Hu, Ri and CV-2 antibodies [29]. Extensive investigations may be needed to find underlying malignancy including FDG-PET scans. Paraneoplastic neuropathy typically presents as a subacute sensory neuronopathy, but its diverse manifestations also include length-dependent sensorimotor polyneuropathy, mononeuritis multiplex and plexopathies. Symptoms of paraneoplastic sensory neuronopathy usually start with sensory loss, ataxia and neuropathic pain. Immunotherapy is usually not effective, but successful tumor removal may result in an improvement of neurologic symptoms as well. Symptomatic treatment may be needed for neuropathic pain and dysautonomia [29].

#### 1.6. Other Autoimmune Neuropathies

Autoimmune neuropathies may occur as isolated disorders affecting peripheral nerves only or may occur in the context of multisystemic autoimmune conditions (e.g. sarcoidosis).

Multifocal motor neuropathy (MMN) presents with slowly progressive, asymmetric weakness in the territories of single nerves without sensory involvement, more frequently involving the upper extremities. The electrodiagnostic studies should reveal persistent conduction block outside common areas of compression along with other demyelinating features. The protein levels in the cerebrospinal fluid are usually normal, and IgM antibodies to GM1 may be found in the serum of up to 80% of these patients. MMN does not respond well to corticosteroids or plasma exchange, and these treatments may even worsen the condition, but it does respond well to treatment with intravenous immunoglobulin [12].

More recently, a new syndrome has been described in porcine processing plant workers after exposure to aerosolized brain tissue, resulting in polyclonal immune response targeting neural tissues. These patients have presented with painful sensory polyradiculoneuropathy, seizures and encephalopathy [30].

Other autoimmune disorders associated with neuropathy include Sjogren’s syndrome, sarcoidosis, inflammatory bowel disease and celiac sprue [31-35]. Sjogren’s syndrome is most commonly associated with length-dependent painful neuropathy or sensory neuronopathy [31, 32]. Some patients may improve with corticosteroids or with cytotoxic medications [31]. Neurosarcoidosis also involves peripheral nervous system and manifests with diverse neuropathy phenotypes [35]. Sarcoid neuropathy most frequently presents as polyradiculoneuropathy or polyneuropathy with frequent cranial nerve palsies. Most patients improve with corticosteroids which may be combined or substituted with cytotoxic medications and TNF antagonists [34, 35]. Autoimmune small fiber neuropathy with dysautonomia may be associated with elevated titers of anti-ganglionic acetylcholine receptor antibodies [36].

### 2. Autoimmune Myopathies

Idiopathic inflammatory myopathies are chronic muscle disorders of unknown etiology characterized by the presence of inflammatory muscle infiltrates. Inflammatory myopathies may also occur also with systemic inflammatory disorders and other organ systems may be involved as well. Based on histopathologic features IIM were divided into: (a) polymyositis, (b) dermatomyositis, (c) immune-mediated necrotizing myopathy, and (d) inclusion body myositis [1]. Pathophysiology of idiopathic inflammatory myopathies is still not well understood, but the molecular profiles of inflammatory myopathies are distinct and further research studies may allow us to improve our diagnostic and management strategies [37]. Electromyography is helpful in demonstrating active myopathic disorder, but does not allow distinguishing an inflammatory myopathy from muscular dystrophy and tissue diagnosis remains the “gold standard”. Utility of autoantibodies in the evaluation of patients with inflammatory myopathies remains mostly unclear although some may have a predictive value for development of certain type of complications (e.g. Jo1 - interstitial lung disease). Increased risk of cancer has been found mostly in patients with dermatomyositis, and to the lesser extent in polymyositis and necrotizing myopathy.

#### 2.1. Polymyositis

Polymyositis typically affects adult patients and manifests with predominant proximal symmetric weakness. Laboratory tests typically show elevated CK, up to 50X normal values. Elevated titers of anti-Jo1 antibodies are associated with interstitial lung disease and anti-synthetase syndrome. Some patients may also develop myocarditis. Weakness predominantly affects proximal muscles. Histopathologic studies in polymyositis typically show endomysial inflammation, but similar findings may be also seen in IBM and a variety of muscular dystrophies. Corticosteroids are usually considered the first choice in the treatment [1]. Second-line agents include IVIG, steroid-sparing cytotoxic medications, biologicals and plasmapheresis.

#### 2.2. Dermatomyositis

Dermatomyositis is usually associated with typical skin rash which may precede or concur with onset of muscle weakness. Less commonly, the rash may lag the onset of weakness. Histopathological studies show perifascicular atrophy with inflammatory infiltrate containing CD4+ cells. In addition to skeletal muscle inflammation, interstitial lung disease and myocarditis may occur as well, similarly as in polymyositis. The presence of anti-Mi2 antibodies is associated with an abrupt onset and good response to therapy. Dermatomyositis may affect both children and adults, while other inflammatory myopathies typically affect adult patients. Weakness predominantly affects proximal muscles, similarly as in polymyositis. Treatment is usually based on the use of corticosteroids. Second-line agents include IVIG, steroid-sparing cytotoxic medications (azathioprine, methotrexate), biologicals and plasmapheresis. Methotrexate is avoided in patients with interstitial lung disease or anti-Jo1 antibodies. Dermatomyositis is also associated with an increased risk of different types of cancer [38].

#### 2.3. Necrotizing Myopathy

Autoimmune necrotizing myopathy can be associated with rapidly progressive weakness, high CPK and little inflammation on muscle biopsies [39]. Histopathologic studies show necrotic myofibers and thickened blood vessels with few white blood cells. Serology may show elevated anti-SRP antibodies, and CPK is usually fairly high (above 10 times the limit of normal) (Table **2**). Some patients may improve with corticosteroids. Paraneoplastic necrotizing myopathy can also be associated with different types of cancer [40].

#### 2.4. Inclusion Body Myositis (IBM)

Sporadic IBM is the commonest of the inflammatory myopathies above the age of 50. It combines features of an inflammatory and neurodegenerative disorders and is usually fairly resistant to treatment [41]. Histopathologic studies show many myofibers with rimmed vacuoles and myofibers surrounded by inflammatory cells. Clinically, patients frequently present with forearm flexor and quadriceps weakness, and bulbar involvement is also common. Slow progression is usually not affected by immunosuppressive or other treatments, but recent study suggested potential usefulness of anti-CD52 monoclonal antibody alemtuzumab [42].

#### 2.5. Other Autoimmune Myopathies

Other inflammatory autoimmune muscle disorders include eosinophilic myositis and perimyositis and different overlap syndromes combining a connective tissue disorder and myositis (e.g. lupus myositis). Treatment is usually similar as with polymyositis/dermatomyositis or is based on the treatment of underlying connective tissue disorders.

### 3. Neuromuscular Junction Disorders

The neuromuscular junction may also be affected by autoimmune processes affecting presynaptic and postsynaptic function. Most common is acquired myasthenia gravis, while Lambert-Eaton myasthenic syndrome remains fairly rare.

#### 3.1. Acquired Myasthenia Gravis

Acquired myasthenia gravis is a prototypic antibody-mediated autoimmune disorder with a reported annual incidence of 3-4/1,000,000 population. Women are affected more than men during childbearing years, and trend reverses after the age of 50 [43]. The clinical hallmarks are fluctuating weakness and muscle fatigue. Among the presenting symptoms are diplopia, ptosis, dysphagia, dysarthria, facial weakness, and shortness of breath. The muscle weakness is usually worse after repeated activity and improves with rest.

Acquired myasthenia gravis is one of the most studied human autoimmune diseases and animal model of experimental autoimmune myasthenia helped us to elucidate its autoimmune mechanisms [44]. The autoantibodies in myasthenia gravis result in loss and dysfunction of the acetylcholine receptors on the post-synaptic muscle membrane, and eventually transmission failure which leads to the clinical symptoms. Antibodies against the acetylcholine receptor (AChR) are detected in up to 85% of patient with generalized disease and 50% of patients with ocular myasthenia gravis [3]. Autoimmune myasthenia is associated with frequent abnormalities of thymus, especially thymic hyperplasia, and 10-15% of patients may have a thymic tumor [45]. More recently, elevated titers of antibodies targeting muscle-specific kinase (MuSK) have been described in almost half of the “seronegative” patients [46]. The patients with MuSK-positive myasthenia frequently have significant selective facial, bulbar, or neck weakness. They may also have muscle atrophy and their symptoms may not be helped by cholinesterase inhibitors.

Acquired myasthenia gravis is typically treated by corticosteroids, commonly used in combination with steroid-sparing medications (azathioprine, mycophenolate) [3, 47]. Maintenance therapy with corticosteroids may be initially complicated by transient exacerbation of symptoms. Corticosteroids may be started with a higher dose from the very beginning (starting prednisone at 0.75- 1 mg/kg/day, then gradually tapering down by 5-10 mg every 4-8 weeks), and then tapered down, but this may be associated with more severe transient exacerbation. Alternatively, initial low dose of corticosteroids may be gradually escalated until target dose is reached (starting prednisone at 10-25 mg daily and gradually increasing to 60-100 mg on alternate days). Clinical studies showed similar effectiveness of azathioprine and corticosteroids, and azathioprine is usually used to lower the dose of corticosteroids [48]. Another purine antagonist, mycophenolate is also frequently used in the treatment of myasthenia. The role of mycophenolate in the treatment of myasthenia gravis has not been definitely established as initial study results are not very encouraging [49]. Other cytotoxic medications used as steroid-sparing agents or monotherapy in myasthenia include tacrolimus, cyclosporine and methotrexate [50]. Other option usually used for induction therapy or treatment of exacerbations include IVIG and plasma exchange. Thymectomy is also sometimes performed empirically in younger myasthenic patients, but the benefit of thymus removal in nonthymomatous myasthenia remains uncertain [51]. "Seronegative" myasthenia associated with anti-MuSK antibodies also responds to immunotherapy with most patients improving with corticosteroids and plasma exchange, but IVIG does not seem to be as effective [52]. Immunotherapy in myasthenia is supplemented with symptomatic treatment of neuromuscular transmission deficiency with cholinesterase inhibitors (pyridostigmine). More recently, experimental studies showed benefits of antisense treatment with an oligopeptide targeting acetylcholinesterase readthrough transcript, but this medication has not been approved yet in USA [53].

##### 3.1.1. Myasthenic Crisis

The course of myasthenia is fairly heterogeneous, and  up to one fifth of all patients will experience a crisis with  respiratory failure and inability to manage secretions during  their lifetime. The crisis is often precipitated by an event  such as infection, trauma, or use of medications such as  aminoglycosides, quinolones, magnesium, or high dose steroids [54]. Treatment of myasthenic crisis is based on the combination of aggressive immunotherapy and adequate supportive measures including invasive mechanical ventilation if needed. Plasma exchange is usually a preferred treatment with its rapid response, and IVIG may be helpful as well as an alternative treatment [3, 54]. As effects of plasma exchange are only temporary, maintenance immunotherapy needs to be adjusted as well.

##### 3.1.2. Ocular Myasthenia

Most myasthenic patients eventually develop ocular symptoms and up to 17% have exclusive ocular myasthenia which may generalize within 2 years from the onset of symptoms [3]. The use of immunosuppressive treatment for ocular myasthenia has been somewhat controversial given the usually benign clinical presentation and potential side effects. However there is evidence that corticosteroids may effectively treat the symptoms and reduce the rate of transformation into generalized myasthenia gravis [55].

#### 3.2. Lambert Eaton Myasthenic Syndrome

Lambert-Eaton myasthenic syndrome is presynaptic neuromuscular junction disorder associated with elevated titers of anti-VGCC antibodies. It frequently occurs in patients with small cell lung carcinoma and newly diagnosed LEMS requires an aggressive evaluation for possible malignancy. However, in 30% of patients there is no evidence of cancer. Autoantibodies targeting SOX1 antigen have been reported as a marker of paraneoplastic LEMS [56]. In some patients with paraneoplastic LEMS, treatment of cancer may also result in improved symptoms of LEMS. Symptomatic treatment with potassium channel blocker 3,4-diaminopyridine leads to improvement in more than 85% of patients with LEMS [57], but the medication is still not approved for clinical use in US. Other options for symptomatic treatment include guanidine and pyridostigmine [58]. Immunomodulatory treatment with IVIG, plasma exchange, corticosteroids and azathioprine may be helpful in some treatment resistant patients, but the extent of improvement is usually limited.

### 4. Autoantibodies

As a part of an abnormal autoimmune response, autoantibodies are frequently found in sera of patients with autoimmune neuromuscular disorders. Many of these antibodies are non-specific and are encountered in other autoimmune conditions (e.g. antinuclear antibodies; ANA), but some are relatively specific for individual neuromuscular conditions (Table **2**). However, the exact role remains uncertain for most autoantibodies associated with autoimmune neuromuscular disorders. It is unclear whether antibodies play an active role in the process or are just markers useful for diagnosis. 

## B. THERAPY

Treatment of autoimmune neuromuscular disorders is largely based on the use of corticosteroids, IVIG or plasma exchange (Table **3**). Other immunosuppressive medications may be used as well, alone or more commonly in combination with corticosteroids or other IVIG/plasma exchange. Long term use of immunosuppressive medications has been associate with an increased risk of malignancies (cyclophosphamide – bladder carcinoma, azathioprine – hematologic malignancies) and opportunistic infections (rituximab - progressive multifocal leukoencephalopathy). Therefore, patients should be treated with the lowest effective dose to reduce treatment-associated risks. Controlled trials have been completed in various neuromuscular disorders, including GBS, CIDP, myasthenia, and inflammatory myopathies, but we still do not have a consensus on most treatment aspects. Choice of therapy in individual cases should be based on treatment efficacy, individual clinical features, and risks of complications.

### 1. Corticosteroids

Corticosteroids have now been a mainstay of treatment of autoimmune and inflammatory conditions for several decades. Different corticosteroid compounds show different immunosuppressive, glucocorticoid and mineralocorticoid potency. Immunosuppressive and anti-inflammatory effects of corticosteroids are related to the impact on concentration, distribution and functions of white blood cells mostly mediated through transcriptional regulation, and primarily affect cell-mediated immunity. Side-effects associates with short- and long-term use of corticosteroids include: weight gain, cosmetic effects (e.g. hirsutism, moon facies), dysthymia, osteoporosis, glucose intolerance, dyspepsia, cataracts, tremor and hypokalemia [59]. Prolonged use of corticosteroids may also lead to steroid myopathy. Various corticosteroid preparations are used orally or intravenously with a regular dosing regimen or with pulse treatments (e.g. 5 day course of high dose intravenous methylprednisolone or dexamethasone). Protocols for dosing are not uniformly accepted and are mostly starting with high dose of prednisone at 0.75-1.5 mg/kg/day used for 2-6 weeks, then tapered down to every other day regimen. Tapering schedule is dependent on patient's symptoms, and the dose of prednisone may be reduced by 5-10 mg mg/day every 1-4 weeks. Typically, we are aiming for alternate day dosing with maintenance therapy, and use steroid-sparing agents to reduce corticosteroid dose (e.g. azathioprine, mycophenolate). In myasthenia gravis, there may be a transient worsening of symptoms following starts of corticosteroids and initial lower dose with gradual increments may be considered [3].

Corticosteroids are typically used in the treatment of CIDP, vasculitis, polymyositis, dermatomyositis and myasthenia (Table **3**). Their use is not recommended in the treatment of GBS and MMN.

### 2. Cytotoxic Medications

Cytotoxic medications are used in treatment of auto-immune neuromuscular disorders as a monotherapy or as steroid-sparing medications. Most commonly, purine antagonists, DNA-alkylating medications and calcineurin inhibitors are used depending on underlying disease and individual patient’s features and comorbidities. Immunosuppressive mechanisms of cytotoxic medications are complex and affect proliferation and activation of specific populations of leukocytes.

Azathioprine and mycophenolate both act as purine antagonists and inhibit resting (G1) and DNA synthesis (S) phases of cell cycle. Azathioprine is metabolized to 6-mercaptopurine. It is mostly used as a steroid-sparing agent in the treatment of myasthenia gravis, and small randomized studies showed similar efficacy of azathioprine compared to corticosteroids when used as monotherapy [48]. Anecdotal reports suggest it may be helpful in some patients with autoimmune neuropathies and myopathies, but this was not confirmed with clinical studies [15]. Mycophenolate blocks de novo purine synthesis and is mostly used as a less toxic steroid-sparing agent in myasthenia, although its efficacy has not been established with certainty and recent controlled trials did not demonstrate steroid-sparing benefits [49]. While individual patients with peripheral neuropathy may respond to mycophenolate, it is usually considered as a third line agent after other options have failed or are contraindicated.

Cyclophosphamide is a DNA-alkylating drug blocking all phases of cell cycle. It is usually used in the treatment of vasculitis, pretreatment for stem cell transplantation, or as a rescue therapy for severe neuromuscular cases. Benefit must be weighed against risk of bladder carcinoma and bone marrow suppression. Cyclophosphamide is given orally or with pulse intravenous therapy. Paradoxically, rare patients with long-standing chronic autoimmune neuropathies may worsen after treatment with cyclophosphamide [16].

Calcineurin inhibitors, cyclosporine and tacrolimus are less commonly used in the treatment of autoimmune neuromuscular disorders. Inhibition of enzyme calcineurin leads to blockade of T cell activation. Recent studies provide encouraging data on potential use of tacrolimus in myasthenia gravis, [50] but more definite proof of its efficacy is still needed especially given the potential nephro- and neurotoxicity. There are also anecdotal reports of the effectiveness of cyclosporine in the treatment of refractory CIDP and MMN [60].

Other less commonly used agents include methotrexate, chlorambucil and fludarabine.

### 3. Plasma Exchange

Plasma exchange is a well established procedure in the treatment of autoimmune disorders. Mechanisms of action of therapeutic plasmapheresis are still not well understood, and may include removal of antibodies, immune complexes, toxins or cytokines. Complications associated with plasmapheresis include hypotension, hypocalcemia and complications associated with maintenance of intravenous access. Its therapeutic role has been established in the treatment of Guillain Barre syndrome, CIDP, paraproteinemic neuropathy, myasthenia gravis and Lambert Eaton myasthenic syndrome [61, 62]. Plasmapheresis may be also considered in treatment of patients with acquired neuromyotonia, stiff-man syndrome, and cryoglobulinemic polyneuropathy, but these decisions should be made on a case-by-case basis. Plasmapheresis has no role in treating patients with ALS or paraneoplastic syndromes with circulating autoantibodies, and should not be combined with IVIG.

### 4. Intravenous Immunoglobulins (IVIG)

IVIG represents a conglomerate of purified immunoglobulins obtained from a pool of several thousands of donors. The predominant components are IgG immunoglobulins and different products vary by IgA content, shelf life and purification process. The exact pathophysiology underlying pharmacologic effects of IVIG remains unclear, but proposed mechanisms include: neutralization of autoantibodies, saturation of Fcgamma receptors and inhibition of complement activation [63]. Use of IVIG is recommended in the treatment of GBS, CIDP, MMN, myasthenia gravis, LEMS, dermatomyositis and stiff person syndrome [64]. Recent studies suggest potential subcutaneous delivery of IVIG may work as well as intravenous [65]. Use of IVIG is associated with an increased risk of stroke and other thromboembolic events, aseptic meningitis and fluid overload [66]. Patients with IgA deficiency may also develop hypersensitivity reaction when given IVIG.

Typical protocol includes the use of IVIG at 2 grams per kilogram divided over 3-5 days, and depending on treated condition and individual patient’s characteristics, maintenance protocol may include monthly infusions of IVIG starting at 0.5-1 g/kg. Half-life of IVIG usually is estimated at 18 to 32 days leading to monthly maintenance regimens [63].

### 5. Other Biological Agents

Monoclonal antibodies targeting B- and T-lymphocytes have been used in the treatment of AND. Additionally, other "biological" medications, including anti-TNF agents have been described both as potential treatments and triggers of autoimmune neuromuscular disorders. Anti-CD20 chimeric antibody rituximab is approved for treatment of lymphoma and rheumatoid arthritis. It has been mostly used off-label in the treatment of IgM paraproteinemic neuropathy, but there are also few reports of its efficacy in treatment resistant CIDP and MG, and results of NIH study of its use in polymyositis are pending [23, 24]. Anti-CD52 antibody alemtuzumab which primarily depletes T lymphocytes showed promise in the treatment of IBM and it may be potentially useful in the treatment of refractory CIDP [42, 67]. Few reports suggest TNF-alpha antagonists etanercept and infliximab may be helpful in the treatment of inflammatory myopathies and myasthenia, but these medication can also precipitate new onset of autoimmune neuropathy [68]. While initial reports suggested interferon-alpha and interferon-beta as potential options in the treatment of CIDP, recent placebo-controlled study with interferon-beta-1a was negative [69].

### 6. Stem Cell Transplantation

Stem cell transplantation in combination with myeloablative and nonmyeloablative chemotherapy has been used to reboot the immune system in the treatment of refractory autoimmune neuromuscular disorders with variable success [70]. Its wider use has been limited by significant morbidity associated with the procedure.

### 7. Other Treatments

#### a. Thymectomy

The role of thymectomy in the treatment of myasthenia gravis is not established but it is common clinical practice to refer myasthenic patients for thymectomy in the absence of thymoma, especially the younger patients. Typically, thymectomy is considered as a therapeutic option in anti-AChR-positive, generalized MG with disease onset before the age of 50 years. The most common histopathologic finding is thymic hyperplasia, but thymoma has been reported in up to 10-15% of patients with myasthenia. Surgical treatment of thymoma is standard in patients with myasthenia or without it, especially since few patients have malignant form and may need more extensive treatment including irradiation and chemotherapy. Reports of efficacy of thymectomy in the treatment of myasthenia are mostly anecdotal and thymectomy is considered only as an option for nonthymomatous myasthenia [51]. The results of a large international single-blinded randomized trial are pending at this time. Thymoma has been also reported in other autoimmune neuromuscular disorders including myositis, autonomic neuropathy, neuromyotonia and stiff person syndrome [71]. At this time, the only absolute indication for thymectomy is the removal of thymoma.

#### b. Total Lymphoid Irradiation

Total lymphoid irradiation has been used as a rescue therapy in the past for severe myasthenia gravis, autoimmune neuropathies and for amyotrophic lateral sclerosis with mostly little or no success and its use is generally not recommended at this time in the treatment of autoimmune neuromuscular disorders.

## Figures and Tables

**Table 1.** Common Postinfectious and Paraneoplastic Neuromuscular Disorders

**Postinfectious**
*Neuropathies *- GBS (common), CIDP (less common)
**Paraneoplastic**
*Neuropathies* - Sensory neuronopathy, autonomic neuropathy
*Myopathies* - polymyositis, dermatomyositis, necrotizing myopathy
*Neuromuscular Junction disorders* - myasthenia gravis, LEMS
*Other* - stiff person syndrome, neuromyotonia

**Table 2.** Auto Antibodies Associated with Autoimmune Neuromuscular Disorders and Clinical Phenotype

Antibodies	Clinical Phenotype
Anti-GM1 antibodies	Multifocal motor neuropathy
Anti-GQ1b antibodies	Miller-Fisher syndrome
Anti-MAG antibodies	Distal acquired demyelinating sensory and motor neuropathy
Anti-ganglionic AChR (nicotinic) antibodies -	Dysautonomic neuropathy
Anti-AChR (nicotinic) antibodies-	Myasthenia gravis
Anti-MuSK antibodies-	“Seronegative” myasthenia gravis
Anti-VGCC antibodies-	Lambert-Eaton myasthenic syndrome
Anti-VGKC antibodies-	Neuromyotonia
Anti-GAD65 antibodies-	Stiff person syndrome
Anti-Amphiphysin antibodies-	Stiff person syndrome
Anti- Jo1 antibodies-	Anti-synthetase syndrome
Anti-Mi2 antibodies	Classic Dermatomyositis with good treatment response
Anti-SRP antibodies-	Treatment resistant, necrotizing myopathy
Anti-Hu Antibodies	Sensory neuronopathy

**Table 3.** Treatment Options for Therapy of Autoimmune Neuromuscular Disorders

	*Corticosteroids*	*IVIG*	*Plasma exchange*	*Azathioprine*	*Cyclophosphamide*	*Mycophenolate*	*Rituximab*
CIDP	1	1	2	2	3	3	3
GBS	-	1	1	-	-	-	-
Multifocal motor neuropathy	no	1	2 (with CYC)	-	2 (also with PLEX)	-	3
Vasculitic neuropathy	1	2	2	3	1	*	*
Anti-MAG	-	-	-	-	-	-	*
Polymyositis	1	2	*	2	*	3	*
Dermatomyositis	1	2	*	2	*	3	*
Inclusion body myositis	-	*	-	-	-	-	-
Myasthenia gravis	1	Cr,^m^	Cr,^m^	m	*	m	*

Anti-MAG - neuropathy associated with anti-MAG antibodies

1 first option

2 second option

3 third option

* may consider in individual cases

cr- treatment of myasthenic crisis

m maintenance treatment

no should avoid.
